# Cultural bias in large language models’ ability to follow neuroradiology guidelines

**DOI:** 10.1007/s00330-026-12634-0

**Published:** 2026-05-26

**Authors:** Naël Bazerbachi, Raphaël Bentegeac, Giada Pistilli, Su-Hwan Kim, Philippe Amouyel, Jean-Pierre Pruvo, Lotfi Hacein-Bey, Aghiles Hamroun, Grégory Kuchcinski, Bastien Le Guellec

**Affiliations:** 1 Neuroradiology Department, Lille University Hospital, Lille, France; 2 UMR1167 RID-AGE, Pasteur Institute of Lille, Inserm, Lille University, Lille University Hospital Center, Lille, France; 3 Department of Public Health and Epidemiology, Lille University Hospital, Lille University, Lille, France; 4 UMR 8011 - Sciences Normes Démocratie (SND), Sorbonne Université, CNRS, Paris, France; 5 Radiology Department, Technical University Munich, Munich, Germany; 6 INSERM, U1172-LilNCog-Lille Neuroscience & Cognition, Université de Lille, Lille, France; 7 Radiology Department, Stanford School of Medicine, Palo Alto, CA USA

**Keywords:** Artificial intelligence, ChatGPT, DeepSeek, Geographic bias, Mistral

## Abstract

**Objectives:**

Large language models (LLMs) are increasingly explored as decision-support tools in medical imaging. However, their ability to align with country-specific guidelines, which often diverge, remains uncertain. We set out to evaluate the geographic neutrality of three state-of-the-art LLMs—GPT-o3, Mistral Large, and DeepSeek R1—and a biomedical LLM (MedGemma 1.5 4B), when applied to neuroradiology scenarios with conflicting U.S. and non-U.S. recommendations.

**Materials and methods:**

Vignettes derived from contradictory international guidelines were presented to each model under two conditions: an implicit setting, where no guideline was specified and vignettes were provided in English and French; and an explicit setting, where prompts directed models to follow a named guideline. Performance was reviewed against the target guideline, and mitigation strategies were tested.

**Results:**

Thirty clinical vignettes presenting conflicting guidelines were evaluated by GPT-o3, Mistral Large, and DeepSeek R1. In the implicit setting, all models favored U.S. guidelines, with GPT-o3, Mistral, and DeepSeek aligning with them in 27 of 30 scenarios (90.0%; 95% CI, 74.4–96.5). In the explicit setting, adherence declined sharply for non-U.S. recommendations for all models. Providing the complete guideline text was the most effective mitigation strategy, restoring accuracies above 90% across all models.

**Conclusion:**

Across languages and model origins, LLMs exhibited a systematic bias toward U.S. neuroradiology guidelines, even when explicitly instructed otherwise. This U.S.-centrism likely reflects training data imbalances and raises concerns for safe global deployment. Strategies for local contextualization, such as guideline integration at deployment, are necessary to ensure context-appropriate clinical decision support.

**Key Points:**

***Question*** *Do large language models display geographical neutrality in neuroradiology decision support?*

***Findings*** *Even models developed in France and China systematically preferred United States guidelines, aligning with them in most implicit scenarios while failing to follow explicit guidelines from other sources.*

***Clinical relevance*** *This systematic United States-centric bias poses clinical and legal risks for global deployment. Safe implementation requires specific localization strategies, such as providing full guideline texts, to ensure recommendations align with local practice standards.*

**Graphical Abstract:**

**Figure:**
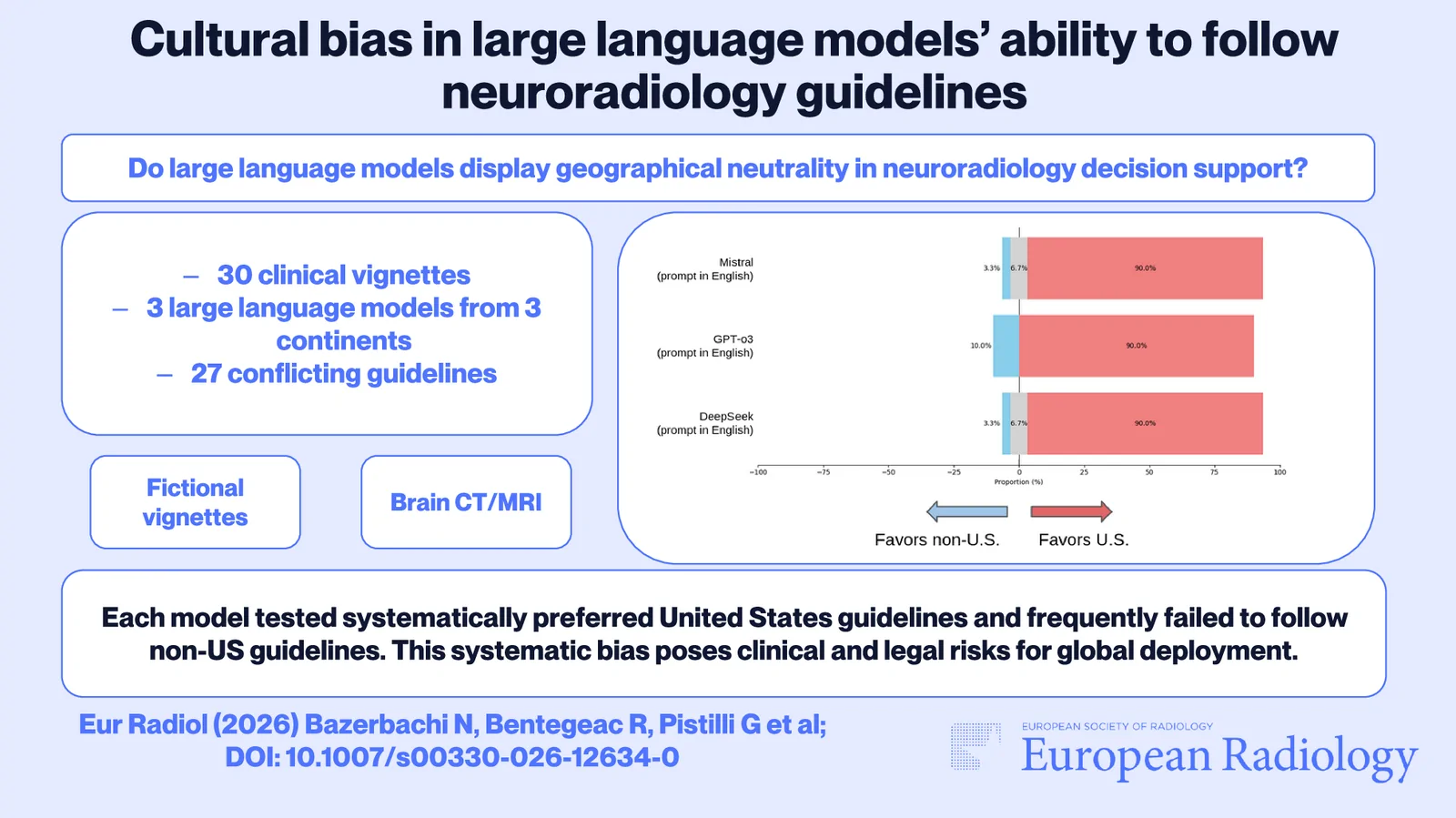

## Introduction

Medical imaging is now deployed across an ever-growing range of highly specialized fields, making its use increasingly complex [1–3]. Because imaging indications are so numerous, clinicians can struggle to order the most appropriate study, and over-utilization of imaging modalities is common [1]. Although professional-society guidelines are regularly issued to guide ordering behavior, their sheer number and frequent updates can make them difficult to navigate [4–6]. A recent multi-country evaluation conducted in Europe revealed significant variations in guideline adherence, ranging from 58% to 86% [7]. Moreover, guidelines issued by societies from different countries can conflict, especially in neuroradiology [8]. Comparative analyses of referral guidelines show only slight concordance between recommendations for common scenarios such as cervical spine imaging [9]. As such, clinicians need a decision-support tool that is adapted to their specific practice context.

Large language models (LLMs) seem promising in this respect, as they can encode extensive clinical knowledge and deploy it to support medical decision-making [10, 11]. In neuroradiology, they have already been tested as imaging decision aids [12].

However, recent studies have uncovered biases in the way LLMs handle medical questions, propagating gender and racial stereotypes [13, 14]. Recent systematic reviews reveal that gender bias occurs in 93.7% of evaluated studies and racial/ethnic bias in 90.9% [15]. While these demographic biases stem from training data imbalances and can perpetuate health disparities, geographic bias, defined as the systematic preference for one country’s clinical guidelines over equally valid alternatives, remains largely unexplored. Unlike demographic bias, which affects individual patient recommendations, geographic bias threatens to misalign entire clinical workflows with local practice standards, potentially disrupting integrated care pathways that reflect region-specific resource availability, treatment philosophies, and regulatory frameworks.

Several studies report a tendency of LLMs to reproduce U.S.-centric values [14–16], but the extent to which this phenomenon applies to medicine remains unknown. In radiology, bias toward American content may generate inappropriate responses with respect to the practice context of the user, with potential medical and legal consequences. To the best of our knowledge, no study has investigated the existence of geographical bias of LLMs for clinical decision support.

We set out to examine whether three large language models, GPT-o3 (OpenAI), DeepSeek R1 (DeepSeek), and Mistral Large (Mistral AI), each developed on a different continent, and a biomedical LLM (MedGemma 1.5 4B, Google), display U.S.-centric bias in clinical decision support by disproportionately adhering to U.S. neuroradiology guidelines over equally authoritative international ones.

## Materials and methods

The requirements to obtain institutional review board approval and informed consent were waived for this study because the study did not involve real patient data or human subjects.

Reporting followed the TRIPOD-LLM [17] guideline.

### Guidelines

Two neuroradiologists (N.B., 1 year of experience; B.L.G., 5 years of experience) independently reviewed hospital cases to identify representative clinical situations for which formal recommendations were available. To ensure accurate interpretation and relevance to the local clinical context, only guidelines published in English or French were considered. Only scenarios for which at least one U.S. society and one non-U.S. society issued conflicting recommendations were retained. A conflict was defined as any discrepancy regarding (1) the indication for imaging, (2) the preferred modality (e.g., CT, MRI, radiography), (3) recommended timing, or (4) technical protocol. The final corpus comprised 30 guidelines issued by 18 professional societies across five geographical entities (US, France, UK, Canada, and Europe) (Supplementary Table 1).

### Adversarial cases

**Fig. 1:**
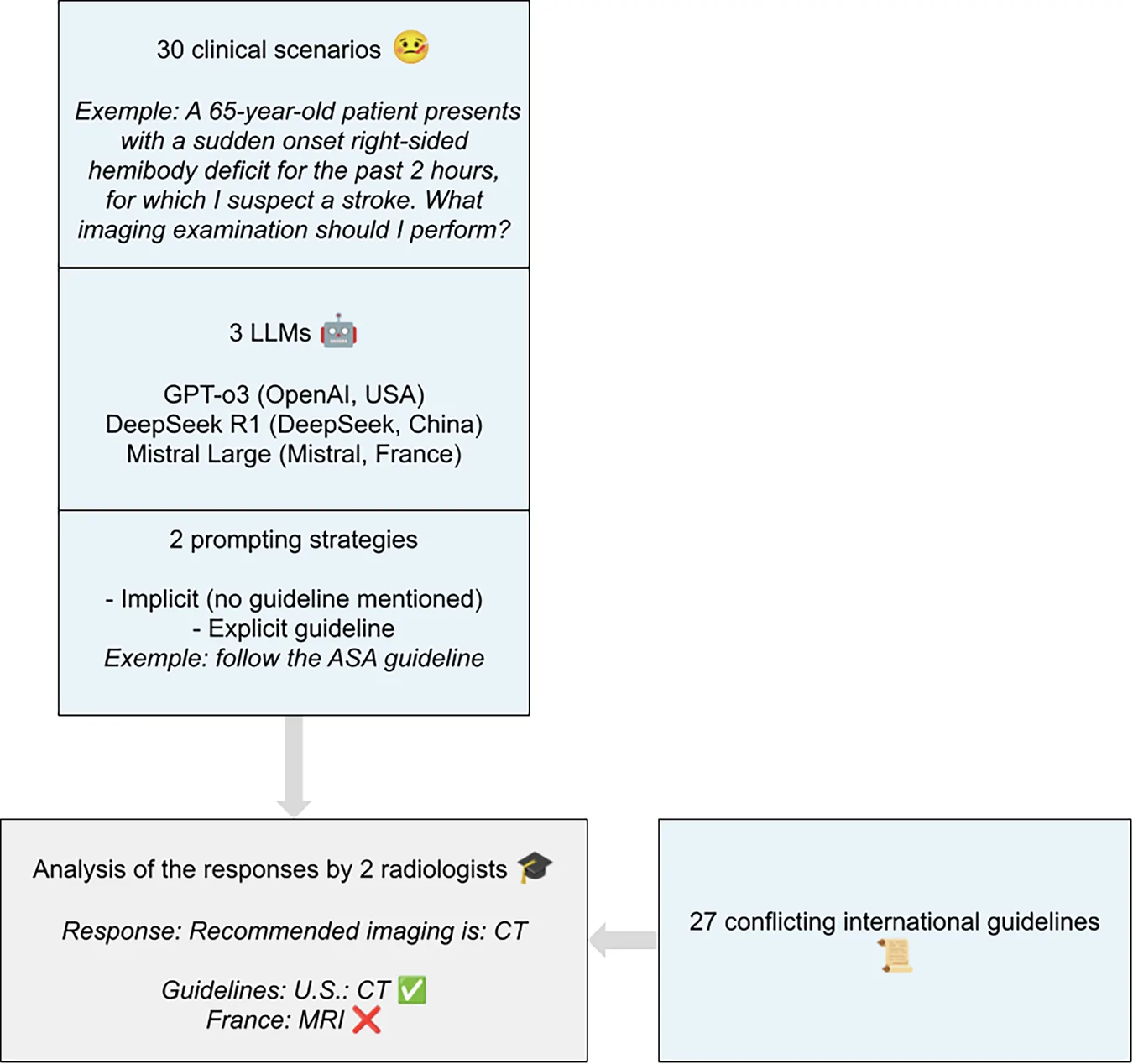
Experimental setup. Large language models were presented with clinical scenarios corresponding to situations with disagreeing guidelines. Models’ responses were analyzed to assess appropriateness relating to the corresponding guidelines

Based on these conflicting guidelines, thirty fictitious clinical vignettes were created. The process was inspired by the authors’ clinical exposure at Lille University Hospital, with anonymisation and adaptation to ensure they remained hypothetical. Each vignette was designed as an adversarial example contrasting U.S. recommendations with one mismatching recommendation from another country (Fig. 1). All vignettes included sufficient information to judge imaging appropriateness, were first written in English, and then translated into French for sensitivity analysis. The complete list of vignettes, themes, and corresponding guidelines is provided in Supplementary Table 2.

### Large language models

Three state-of-the-art large language models were tested: GPT-o3 (model “*o3-2025-04-16*,” OpenAI, U.S.), Mistral Large (model “*mistral-large-2411*,” Mistral AI, France), and DeepSeek R1 (model “*deepseek-reasoner*,” DeepSeek, China). The first two were selected as reference models from the U.S. and France, while DeepSeek was added as a sensitivity analysis to broaden geographical representation. Mistral and DeepSeek models were accessed between March 25th and March 28th, 2025, and GPT-o3 between April 20th and 22nd, through their respective APIs, using default hyperparameters (temperature could not be modified for GPT-o3). In a post hoc analysis, we assessed the MedGemma model (“medgemma-1.5-4b-it,” Google, U.S.), a model specialized in medical images and texts released in January 2026 [18], run on-premise on 3 Quadro RTX Graphical Processing Units with 24 Gb of VRAM each. MedGemma was only assessed on the implicit and explicit settings through a code available at https://github.com/BastienLeGuellec/medgemma-eura, as it natively does not support PDF input or web search. For all models, memory was cleared after each response.

### Prompting

Large language models were queried under two principal conditions. In the implicit‑guideline setting, the prompt presented only the clinical scenario. To probe robustness in the implicit setting, we repeated the queries 24 h later and translated all vignettes into French. In the explicit‑guideline setting, each prompt instructed the model to apply a named recommendation, using this exact phrasing: “[Clinical vignette]. Which imaging should I perform? Answer using the [name of the guideline, for example, New Orleans Head CT Criteria] guideline.” For the explicit‑guideline setting, we also tested two mitigation strategies aimed at improving fidelity: enabling web searches (agentic mode) and providing the complete guideline as a PDF within the prompt context. Those two mitigation strategies were performed directly on the respective model provider website (i.e., chatgpt.com, https://chat.mistral.ai/chat and https://chat.deepseek.com), using the “web search” and “attach document” options to mimic use in a clinical setting. Token counts from each vignette + guideline PDF were below the context limit of all tested models.

### Responses review

Models’ responses were independently reviewed by a neuroradiology trainee (N.B.) and a board-certified neuroradiologist (B.L.G.) for appropriateness relative to each corresponding guideline. Responses containing incorrect exam suggestions, including hallucinated exam names, were treated as failures (not following any guideline). Inter-rater agreement was assessed using Cohen’s kappa. The two raters agreed on 93% of all ratings across implicit, explicit, and mitigation experiments (κ = 0.81, 95% CI [0.78, 0.84]), indicating almost perfect agreement. Discrepancies were resolved through consensus.

### Statistical analyses

Proportions are reported with Wilson 95% confidence intervals. Differences in proportions were compared with two-tailed χ² tests for independent measures and McNemar tests for paired comparisons for the mitigation strategies comparisons. The value of α was adjusted with the Bonferroni method for the primary analysis of implicit and explicit preference of models for US vs non-US guidelines, to a value of α_ajd_ = 0.006 because of multiple testing. For the mitigation strategies, which were exploratory analyses, α was set to 0.05, without correction for multiple comparisons. Analyses were done in Python 3.10 using the scipy module (version 1.14.1).

## Results

### Implicit preference for U.S. guidelines

**Fig. 2:**
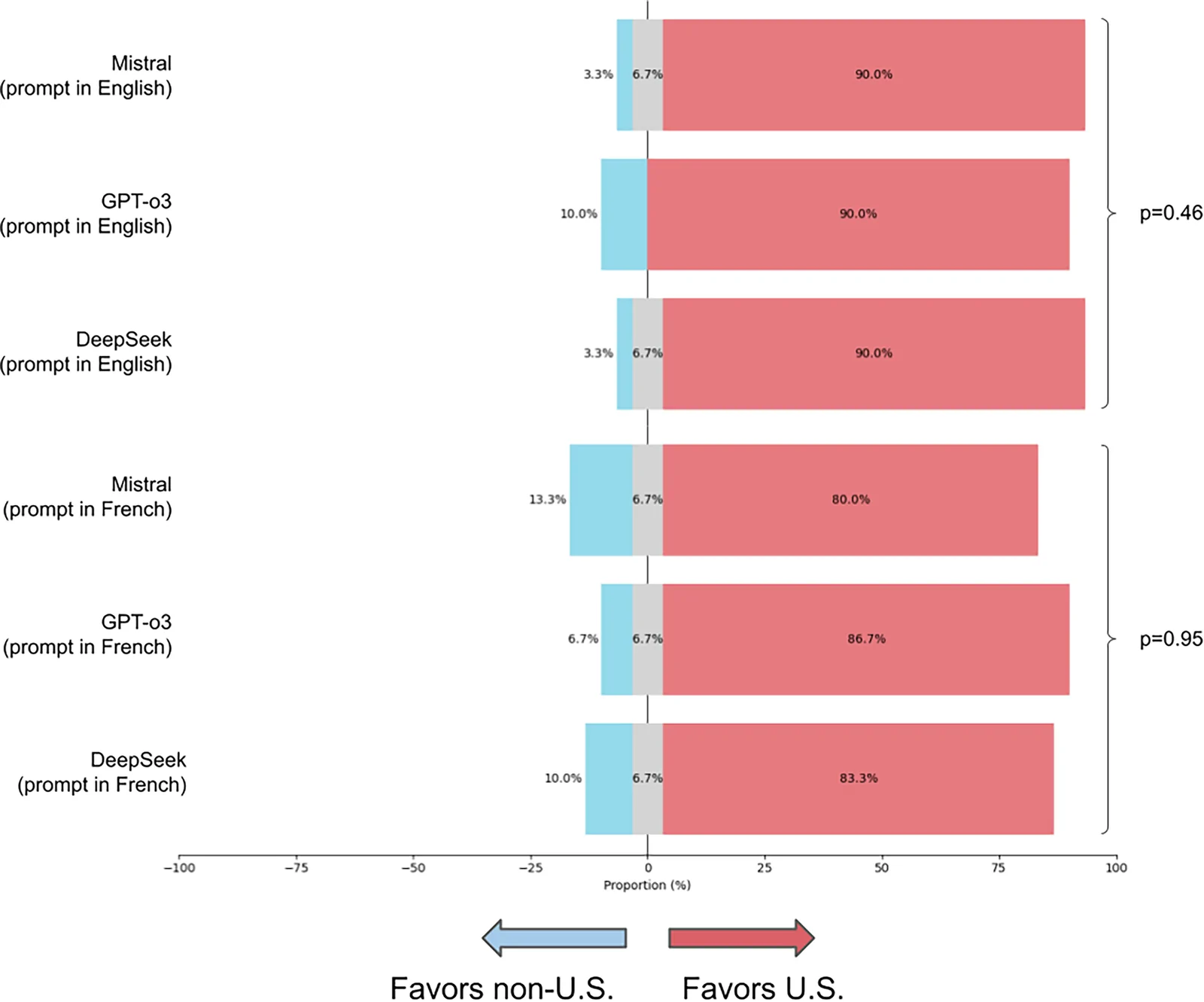
Selection of U.S. vs non-U.S. guidelines in the implicit scenarios, with prompts in English and French

The final analysis included 30 clinical vignettes evaluated by three large language models. In the implicit setting, each model chose the U.S. option more frequently over the non-U.S. option. DeepSeek, GPT-o3, and Mistral sided with the American guideline in 27 of 30 responses (90.0%, 95% CI: 74.4–96.5, all *p* < 0.001) (Fig. 2). There was no statistically significant difference in preference distributions between models (*p* = 0.46). Those results were consistent across 2 runs (Supplementary Fig. 1). Representative examples are presented in Supplementary Table 3.

### The effect of language on the implicit preference

Prompting the model in French did not alter the pattern of U.S. preference. DeepSeek aligned with the U.S. recommendation in 25 of 30 responses (83.3%, 95% CI: 66.4–92.7, *p* < 0.001), GPT-o3 in 26 of 30 responses (86.7%, 95% CI: 70.3–94.7, *p* < 0.001), and Mistral in 24 of 30 responses (80.0%, 95% CI: 62.7–90.5, *p* < 0.001) (Fig. 2). The comparison of preference distributions between models showed no statistically significant difference (*p* = 0.95). Those results were consistent across 2 runs (Supplementary Fig. 1).

### Ability to follow an explicit guideline

**Fig. 3:**
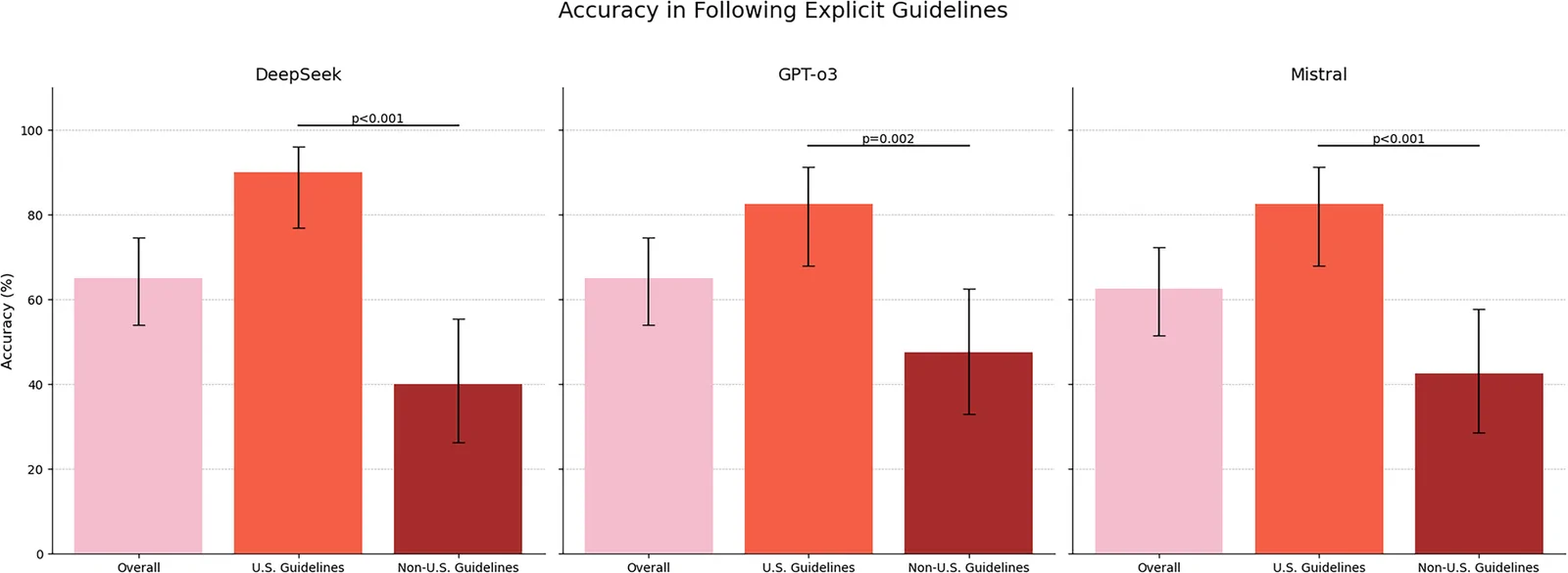
Correct adherence of each model to explicit guidelines, depending on its U.S. vs non-U.S. origin

When models were explicitly instructed to follow a given guideline, their overall accuracy was 58.3% (35/60; 95% CI 45.7–69.9) for DeepSeek, 56.7% (34/60; 95% CI 44.1–68.4) for GPT-o3, and 55.0% (33/60; 95% CI 42.5–66.9) for Mistral (Fig. 3). All three models performed significantly better when the guideline originated from the U.S. than on non-U.S. guidelines. DeepSeek answered 86.7% of U.S. scenarios correctly (26/30; 95% CI 70.3–94.7) versus 30.0% (9/30; 95% CI 16.7–47.9; *p* < 0.001). GPT-o3 answered correctly for 76.7% of vignettes with associated US guidelines (23/30; 95% CI 59.1–88.2) versus 36.7% for vignettes with non-US guidelines (11/30; 95% CI 21.9–54.5; *p* = 0.004). Mistral’s performance showed a similar trend, with 80.0% accuracy on vignettes associated with US guidelines (24/30; 95% CI 62.7–90.5) compared to 30.0% on vignettes associated with non-US guidelines (9/30; 95% CI 16.7–47.9 ; *p* < 0.001).

### Impact of web searches on explicit guideline following

**Fig. 4:**
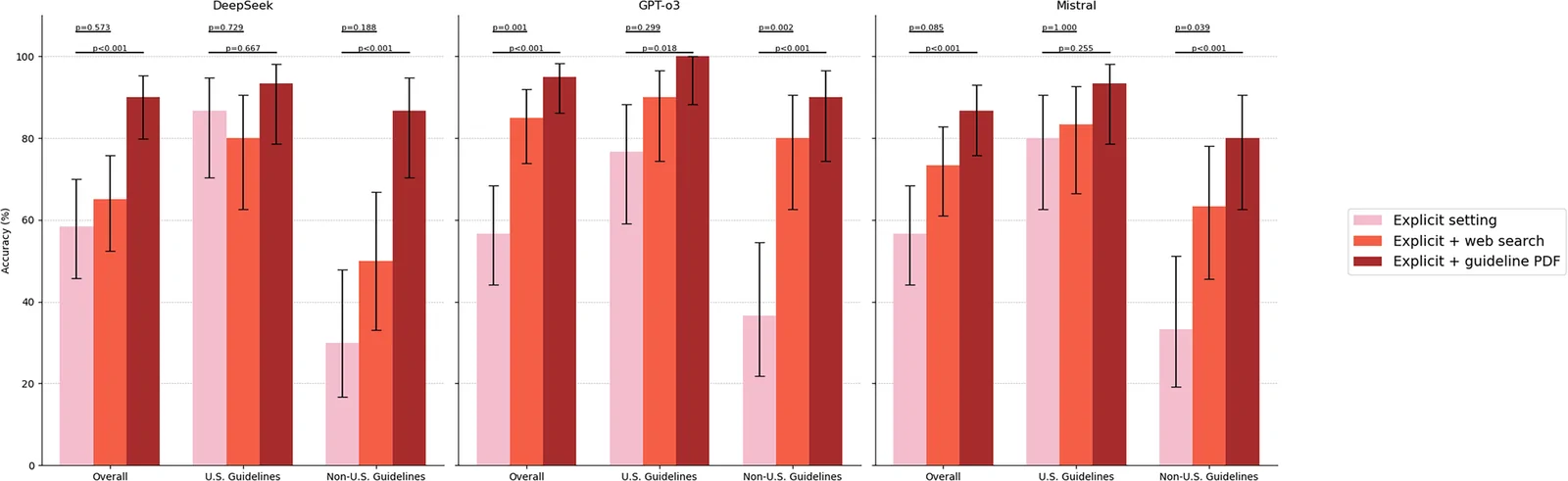
Effect of the mitigation strategies (web search and guideline PDF) on the correct adherence of each model to explicit guidelines, depending on their U.S. vs non-U.S. origin

The web search option improved model adherence to non-U.S. guidelines for GPT-o3 and Mistral but not for DeepSeek (Fig. 4). In this approach, DeepSeek achieved 65.0% overall accuracy (39/60; 95% CI: 52.4–75.8), with 80.0% on U.S. guidelines (24/30; 95% CI: 62.7–90.5) versus 50.0% on non-U.S. guidelines (15/30; 95% CI: 33.2–66.8; *p* = 0.0304). GPT-o3 reached 85.0% overall accuracy (51/60; 95% CI: 73.9–91.9), 90.0% on U.S. guidelines (27/30; 95% CI: 74.4–96.5) versus 80.0% on non-U.S. guidelines (24/30; 95% CI: 62.7–90.5; *p* = 0.4696). Mistral showed a similar pattern, with 73.3% overall accuracy (44/60; 95% CI: 61.0–82.9), with 83.3% accuracy on U.S. guidelines (25/30; 95% CI: 66.4–92.7) versus 63.3% on non-U.S. guidelines (19/30; 95% CI: 45.5–78.1; *p* = 0.1444).

### Impact of providing the guideline as a PDF on explicit guideline following

Providing the guideline as a PDF consistently yielded higher accuracies across all models (Fig. 4). DeepSeek reached 90.0% overall accuracy (54/60; 95% CI: 79.9–95.3), with 93.3% accuracy for U.S. (28/30; 95% CI: 78.7–98.2) versus 86.7% for non-U.S. guidelines (26/30; 95% CI: 70.3–94.7; *p* = 0.6670). GPT achieved 95.0% overall accuracy (57/60; 95% CI: 86.3–98.3), including perfect adherence to U.S. guidelines (100.0%, 30/30; 95% CI: 88.6–100.0) versus 90.0% for non-U.S. (27/30; 95% CI: 74.4–96.5; *p* = 0.2361). Mistral reached 86.7% accuracy overall (52/60; 95% CI: 75.8–93.1), with 93.3% on U.S. (28/30; 95% CI: 78.7–98.2) versus 80.0% on non-U.S. guidelines (24/30; 95% CI: 62.7–90.5; *p* = 0.2546).

### Implicit and explicit preference of MedGemma 1.5

MedGemma demonstrated strong U.S. preference in both English (63.3%, 19/30; 95% CI: 45.5–78.1) and French (60.0%, 18/30; 95% CI: 42.3–75.4, Supplementary Fig. 2), with minimal non-U.S. alignment (0.0% and 6.7%, respectively; both *p* < 0.001). When explicitly instructed to follow named guidelines (Supplementary Fig. 3), MedGemma showed significantly lower accuracy for non-U.S. versus U.S. guidelines in both English (3.3%, 1/30, 95% CI: 0.6–16.7 vs 50.0%, 15/30, 95% CI: 32.6–67.4; *p* < 0.001) and French (16.7%, 5/30, 95% CI: 7.3–33.6 vs 53.3%, 16/30, 95% CI: 35.9–69.9; *p* = 0.007).

## Discussion

Our evaluation of three state-of-the-art LLMs reveals a systematic preference for United States neuroradiology guidelines, even in models conceived and trained on other continents. In the implicit setting, more than 80% of responses aligned with the U.S. guideline rather than the non-U.S. guideline. When the models were told explicitly which guideline to follow, accuracy dropped significantly for non-U.S. recommendations as compared to U.S. ones. This behavior persisted across two prompting languages and three models, underscoring that U.S. centrism is a common trait of current general-purpose LLMs.

Our findings on geographic bias complement but differ importantly from the established literature on demographic bias in medical AI [15, 19]. While demographic biases manifest as differential treatment recommendations for individual patients based on race, gender, or socioeconomic status, geographic bias operates at a systems level, defaulting to one jurisdiction’s clinical protocols regardless of the user’s location. Both bias types share a common origin in training data imbalances [20, 21], but their clinical implications diverge. Demographic bias risks exacerbating existing health disparities within a population, whereas geographic bias risks importing inappropriate care standards across populations [15].

While there is only limited research on geographic bias in medical LLMs, the existing literature supports our findings. A recent European benchmarking study evaluated GPT-4, DeepSeek R1, and Gemini 2.0 against ESGO/ESTRO/ESP guidelines for cervical cancer and found suboptimal accuracy and significant variability in adherence to these European guidelines, underscoring the need for expert oversight to prevent misinformation in European practice settings [22]. This aligns with our observation that LLMs struggle to follow non-U.S. guidelines even when explicitly instructed.

The principal driver of this U.S. centrism is almost certainly data imbalance during training [16]. Even though the training corpus of current models (even open-weights) is confidential, previous iterations of GPT revealed a strong skew toward English texts, with 93% of the texts presented in the models being written in English [16]. In medicine, English literature vastly outweighs material in other languages, and U.S. sources dominate that corpus [23]. A recent study on 77 lung cancer guidelines showed the predominance of U.S. research articles citations in those guidelines [24]. Across the complete dataset, the U.S. accounted for 30.6% of citations, while every other country included was below 15%, including in their own national guidelines. This citation bias may increase the relative weight of U.S. papers in the LLM training corpus, and thus the bias toward those references. Reinforcement learning from human feedback (RLHF) amplifies the imbalance: many raters are crowd-workers who score answers through a U.S. regulatory lens [25]. Combined, these effects create a training environment in which American standards are the path of least resistance for optimization.

The stricter European regulatory landscape, embodied by the General Data Protection Regulation (GDPR), is also a major factor limiting the dissemination of medical datasets representative of European standards. In a recent review, Wu et al showed that the largest providers of open clinical text datasets were the US and China, with many European countries and languages still in the blind spot of current research [21]. This inequitable regulatory landscape also impacts model testing, most of the available tests and pioneering studies still being performed on standardized English texts, originating mostly from a subset of US hospitals [26]. Finally, it will impact adoption, especially in the context of the recent EU AI Act [27]. Judged as “high-risk” contexts, health applications of LLMs are particularly scrutinized by European regulators, and their clinical adoption in Europe may thus suffer from a lag of several years. As an example, the recently released ChatGPT Health is only available in the US, and European counterparts are still awaited [28].

Interestingly, the preference toward US guidelines was present not only in the American-developed GPT-o3 but also in DeepSeek R1 (China) and Mistral Large (France), indicating that the skew is learned from the global data ecosystem rather than imposed solely by U.S. engineers or alignment teams. In other words, the bias appears to be baked into the shared textual substrate on which all three models rely, regardless of the continent where the final weights were tuned.

The clinical stakes of this bias are non-trivial. Divergent imaging indications between jurisdictions reflect trade-offs in radiation exposure, cost, diagnostic yield, and time-to-treatment [29]. In acute stroke, for example, CT and MRI each have distinct advantages and limitations and cannot be interchanged without consequence. Imaging choices are embedded within broader care pathways that encompass emergency logistics, treatment timelines, and resource availability [30]. They reflect underlying treatment philosophies that differ across health systems: for instance, for acute stroke evaluation, U.S. protocols prioritize rapid rule-out of hemorrhage with CT to enable thrombolysis [31], whereas French and Korean pathways emphasize early MRI to guide etiological diagnosis and outcome prediction [32]. Consequently, a model that defaults to the “wrong” modality risks disrupting an integrated workflow rather than simply choosing a suboptimal test.

These discrepancies also raise medico-legal concerns. In France, malpractice standards hinge on what a “reasonable clinician working in France” would do, not on what an algorithm trained in California recommends [33].

Technical mitigations are plausible but undeveloped. Curating multilingual, region-balanced corpora would reduce the dominance of U.S. sources at the training stage [34, 35]. During alignment, developers could include raters from more diverse regulatory lenses and stratify human raters by geography or explicitly instruct them to privilege a wider spectrum of norms when scoring responses. However, implementing this at scale would probably generate prohibitive costs and logistical complexity of sourcing region-specific medical experts rather than generalist crowd-workers. Local, post hoc mitigation strategies may therefore be more adapted. We tested two post-deployment mitigation strategies with beneficial effects: letting LLMs search the web and providing them with the guidelines in PDF. Prompting AIs with the relevant recommendation revealed the most promising. However, even when provided with the correct guidelines, most LLMs did not achieve 100% accuracy. This may be the result of an imperfect PDF file handling by the models’ providers, which probably leverages OCR (optical character recognition), or from difficulty finding the relevant information in potentially large texts (needle in a haystack problem). Ultimately, safe deployments may require country- or hospital-specific systems that fuse foundation-model reasoning with modular policy layers encoding national guidelines.

While our study highlights the specific issue of geographic bias in radiology, this phenomenon is part of the broader, well-documented challenge of medical “concept drift,” where LLMs encounter knowledge conflicts due to differing regional guidelines or the temporal evolution of medical recommendations. Recent natural language processing research evaluates frameworks such as DriftMedQA to systematically assess how medical LLMs maintain reliability under distribution shifts. Traditional alignment techniques, such as reinforcement learning from human feedback (RLHF), are fundamentally limited in resolving these conflicts if the human evaluators inherently apply a U.S.-centric regulatory lens. To actively mitigate these conflicts, researchers are exploring synergistic techniques like retrieval-augmented generation (RAG) combined with advanced fine-tuning methods such as Direct Preference Optimization (DPO) or Odds Ratio Preference Optimization (ORPO) [36]. RAG allows the model to dynamically retrieve up-to-date, localized documents (e.g., a specific European society guideline) prior to generating a response, thereby bypassing the limitations of its static, pre-trained memory. Conversely, preference-based techniques like DPO and ORPO act as direct behavioral coaching; by training the model on contrasting pairs of clinical decisions (e.g., a correct local stroke protocol versus an incorrect U.S.-centric one), these methods explicitly teach the algorithm to consistently prioritize the preferred local standard. As research on resolving data drift progresses rapidly outside of radiology, our findings underscore an urgent need for the radiological community to recognize LLM bias as a dynamic problem. Adapting these advanced alignment frameworks will be essential to ensure the safety, reliability, and contextual appropriateness of AI-assisted clinical workflows worldwide.

One possible solution to access safer clinical decision support could also be the use of domain-specific models. However, our results on MedGemma 1.5 indicate that those models may actually be detrimental to guideline adherence. In line with previous studies, we showed that domain-specialized LLMs performed more poorly on medical tasks as opposed to their generalist counterparts [37]. While all models rarely generated incorrect or hallucinated answers (for example, DeepSeek suggesting a “PERRIA scintigraphy” for brain death), MedGemma had the highest rate of erroneous answers. This may be a result of its significantly smaller size (4B parameter vs 671B for DeepSeek, the second smallest model tested), or the specific fine-tuning that may have altered its instruction-following ability [37]. In addition, it also exhibited the same US preference, which may be a result of its fine-tuning primarily on US biomedical texts, such as MIMIC [18, 21].

We used guideline concordance as the primary metric, which represents an important but incomplete measure of clinical utility. Guideline adherence serves as a reasonable proxy for quality care in the absence of direct outcome data, as clinical practice guidelines are intended to synthesize the best available evidence to optimize patient outcomes. However, the relationship between guideline adherence and patient outcomes is not always linear or consistent across settings [38]. This creates a genuine ethical dilemma when an LLM’s US-biased recommendation might theoretically produce better patient outcomes than no guideline adherence, or even strict adherence to a local guideline. The preliminary evidence from the Penda Health study in Kenya suggests that LLM-based support can reduce diagnostic errors even when not strictly following local guidelines, highlighting that absolute patient benefit and local guideline adherence may sometimes diverge [6]. Ultimately, safe and ethical LLM deployment requires moving beyond the binary of “guideline-concordant” versus “guideline-discordant” to a more nuanced framework that considers context-specific appropriateness, resource availability, and, most importantly, empirical evidence of patient benefit in the local setting [11, 12]. Future research should prioritize pragmatic trials comparing patient outcomes under LLM-assisted care versus conventional care across diverse healthcare systems, stratified by guideline origin and local context.

Our study has limitations. The corpus was confined to neuroradiology, a field whose guideline landscape is unusually rich and sometimes contradictory; other specialties with more homogeneous international standards might show smaller effects. We analyzed only English and French prompts, leaving low-resource languages unexplored. The models tested were snapshots accessed in March 2025. Rapid evolution of AI models could introduce variability in bias profiles and impact the consistency of guideline adherence, warranting the need for ongoing evaluation and transparency regarding AI deployment and its potential to perpetuate or mitigate bias in clinical practice [20]. In addition, we did not test the possible interaction between geographical bias and other biases of LLMs, such as sexual orientation, employment status, or race [13]. Finally, we judged correctness by guideline concordance, not by downstream patient outcomes. Real-world usefulness depends on how well advice improves clinical decisions, a question that will require prospective trials.

We showed that contemporary LLMs carry implicit geographic biases that can misalign with local best practices. As the medical community experiments with these tools for imaging triage and protocols, our findings argue for caution and for robust localization pipelines. More broadly, geographic fairness should join bias metrics such as gender or ethnicity in the standard evaluation battery for clinical AI. Only through deliberate, data-driven mitigation with human-in-the-loop can we ensure that decision support powered by LLMs serves patients everywhere, not just those whose guidelines happen to dominate the training data.

## Supplementary information

ELECTRONIC SUPPLEMENTARY MATERIAL

## Abbreviations

LLM: Large language model
