# Supplementary material for: Cultural bias in large language models’ ability to follow neuroradiology guidelines
Source: Eur Radiol. 2026 May 26;36(10):7444–53. doi: 10.1007/s00330-026-12634-0 (PMC13574872; doi:10.1007/s00330-026-12634-0)
Supplement: Supplementary file 1 [file 330_2026_12634_MOESM1_ESM.pdf]

Cultural bias in large language models' ability to follow neuroradiology guidelines

ELECTRONIC SUPPLEMENTARY MATERIAL

Supplementary Table 1: Theme, origin and society or guidelines included

Theme	Country of origin	Society
Brain Death	France	Agence de la biomédecine
Brain death	USA	AAN
Dementia	France	HAS
Dementia	USA	ACR
Dementia	UK	NHS
Head trauma	Canada	University of British Columbia
Head trauma	France	SFAR
Head trauma	USA	NOC
Head trauma	USA	ACR
Headache	France	SFETD
Headache	France	CEN
Headache	USA	ACR
Infectious	UK	UK Joint Specialist Society Guidelines
Infectious	EU	ESCMID
Infectious	USA	IDSA
Multiple sclerosis	France	OFSEP
Multiple sclerosis	USA	CMSC
Psychiatry	France	SFMU
Psychiatry	USA	ACR
Psychiatry	UK	NICE
Seizure	France	SFN
Seizure	France	HAS
Seizure	USA	ACR
Seizure	UK	NICE

Stroke	France	HAS
Stroke	Canada	Fondation des maladies du cœur et de l'AVC du Canada
Stroke	USA	ASA
Stroke	USA	ACR
Tumor	France	ANOCEF
Tumor	USA	ACR

Supplementary table 2: Clinical vignettes with corresponding theme

A 65-year-old patient presents with a sudden-onset right hemiparesis for the past 2 hours, for which I suspect a stroke. What imaging test should I perform?	Vascular
A 28-year-old female patient with a history of 5 pack-years of smoking and combined estrogen-progestin contraception presents with rapidly progressive headaches in the context of a first generalized seizure, with no prior history of epilepsy, raising suspicion of cerebral venous thrombosis (CVT). What imaging test should I perform?	Vascular
A 35-year-old patient has sustained a head trauma with an initial loss of consciousness. Currently, Glasgow score is 15, and he had a single vomiting episode. Should I perform an imaging test, and if so, which one?	Head trauma
A 35-year-old patient has sustained a head trauma with an initial loss of consciousness. Currently, Glasgow score is 15, and he complains of headaches. Should I perform an imaging test, and if so, which one?	Head trauma
A 35-year-old patient has sustained a head trauma with an initial loss of consciousness in the context of alcohol intoxication. Currently, Glasgow score is 15, with no particular clinical abnormalities. Should I perform an imaging test, and if so, which one?	Head trauma
A 62-year-old patient has sustained a head trauma with an initial loss of consciousness. Currently, Glasgow score is 15, with no particular clinical abnormalities. Should I perform an imaging test, and if so, which one?	Head trauma
A 22-year-old female patient had a first generalized tonic-clonic seizure one hour ago, with no particular medical history and a normal clinical exam. Should I perform an urgent imaging test, and if so, which one?	Epilepsy
A 62-year-old female patient presents with pulsatile tinnitus and a normal clinical exam. What imaging test should I perform?	Tinnitus
A 45-year-old female patient with a history of clinically stable MS, under usual treatment, comes for her annual MRI follow-up. Which MRI sequences should I perform?	Multiple sclerosis

A 38-year-old female patient presents with worsening of her usual headaches, which today do not optimally respond to her regular treatment. Should I perform an imaging test, and if so, which one?	Headache
A 43-year-old female patient presents with a thunderclap headache of sudden onset, maximal intensity immediately. What imaging test should I perform?	Headache
A 30-year-old patient has a clinical diagnosis of brain death with Glasgow 3 coma, abolition of all brainstem reflexes, and absence of spontaneous ventilation on hypercapnia test. Should I perform an additional test, and if so, which one?	Brain death
A 50-year-old patient has an incomplete clinical diagnosis of brain death with Glasgow 3 coma, abolition of all brainstem reflexes, but impossibility to perform the hypercapnia test due to high risk of cardiopulmonary decompensation. Should I perform an additional test, and if so, which one?	Brain death
A 19-year-old female patient presents with a first psychotic episode with delusional ideas and visual hallucinations in the emergency department, with no psychiatric history or medication/alcohol intoxication. Should I perform an imaging test, and if so, which one?	Psychiatry
A 68-year-old patient presents with cognitive decline and episodic memory impairment, suggestive of typical Alzheimer’s disease. Should I perform an imaging test, and if so, which one?	Dementia
A 62-year-old patient presents with cognitive decline and episodic memory impairment, suspicious for Alzheimer’s disease but with some atypical features such as early behavioral changes and early onset. Should I perform an imaging test, and if so, which one?	Dementia
A 55-year-old patient presents with behavioral changes such as disinhibition and hyperorality, associated with progressive cognitive decline, suggestive of frontotemporal dementia (FTD), but with atypical features such as sleep behavior disorder. Should I perform an imaging test, and if so, which one?	Dementia
A 66-year-old patient presents with cognitive decline, visual hallucinations, sleep behavior disorder, without abnormal movements, suggestive of atypical Lewy body dementia. Should I perform an imaging test, and if so, which one?	Dementia
A 63-year-old female patient presents with cognitive decline, urinary symptoms, and gait disturbance suggestive of normal pressure hydrocephalus (NPH). Should I perform an imaging test, and if so, which one?	Dementia
An 80-year-old patient presents with progressive cognitive decline over several years, no longer independent for daily living activities, and residing in a nursing home. Should I perform an imaging test, and if so, which one?	Dementia
A 50-year-old patient, with no medical history, presents with rapidly progressive dementia. Should I perform an imaging test, and if so, which one?	Dementia
A 25-year-old patient presents with fever and meningeal syndrome, without fulminant purpura, Glasgow 10 (Y3V3M4), raising suspicion of infectious meningitis. Should I perform an imaging test before LP, and if so, which one?	Infectious

A 25-year-old patient presents with fever and meningeal syndrome, without fulminant purpura, Glasgow 14, raising suspicion of infectious meningitis. Should I perform an imaging test before LP, and if so, which one?	Infectious
A 25-year-old patient presents with fever and meningeal syndrome, without fulminant purpura, Glasgow 12, raising suspicion of infectious meningitis. Should I perform an imaging test before LP, and if so, which one?	Infectious
A 25-year-old patient presents with fever and meningeal syndrome and papilledema, without fulminant purpura, Glasgow 15, raising suspicion of infectious meningitis. Should I perform an imaging test before LP, and if so, which one?	Infectious
A 25-year-old immunocompromised patient presents with fever and meningeal syndrome, without fulminant purpura, Glasgow 15, raising suspicion of infectious meningitis. Should I perform an imaging test before LP, and if so, which one?	Infectious
A 25-year-old patient with a history of childhood herpes encephalitis presents with fever and meningeal syndrome, without fulminant purpura, Glasgow 15, raising suspicion of infectious meningitis. Should I perform an imaging test before LP, and if so, which one?	Infectious
A 40-year-old patient with a history of right frontal astrocytoma diagnosed 2 years ago presents with fever and meningeal syndrome, without fulminant purpura, Glasgow 15, raising suspicion of infectious meningitis. Should I perform an imaging test before LP, and if so, which one?	Infectious
A 58-year-old patient with a history of superficial right MCA ischemic stroke presents with fever and meningeal syndrome, without fulminant purpura, Glasgow 15, raising suspicion of infectious meningitis. Should I perform an imaging test before LP, and if so, which one?	Infectious
A 45-year-old patient, Glasgow 15, presents with cervical spine trauma without instability or penetrating mechanism, in the context of alcohol intoxication. Should I perform an imaging test, and if so, which one?	Cervical spine trauma

Supplementary Table 3: Representative examples of all models implicitly aligning with the US guideline

Vignette	US guideline	Non-US guideline	DeepSeek	GPT-o3	Mistral
A 65-year-old patient presents with a sudden onset right-sided hemibody deficit for the past 2 hours, for which I suspect a stroke. What imaging examination should I perform?	ASA: non-contrast CT scan	HAS: MRI	In the case of a suspected acute stroke with sudden right-sided hemibody deficits, **immediate neuroimaging** is critical to guide treatment. The recommended imaging approach is **NCCT**	In the case of a suspected stroke with sudden onset hemibody deficits, the initial imaging study of choice is a non-contrast CT	In a case where you suspect a stroke in a 65-year-old patient with a sudden onset right-sided hemibody deficit, Non-Contrast Computed Tomography (CT) Scan of the Head should be performed promptly
A 58-year-old patient, with a history of a superficial right sylvian ischemic stroke, presents with fever associated with a meningeal syndrome, without any indication of fulminant purpura, with a Glasgow score of 15, suggesting infectious meningitis. Should I perform imaging before the lumbar puncture and if so, which one?	IDSA: non-contrast CT	ESCMI: no imaging	Proceed with a **non-contrast CT head** prior to LP.	Yes, perform imaging to rule out raised ICP, mass effect, or other complications. Preferred imaging modality: Non-contrast CT is typically the best first step, as it is quick, effective, and can rule out any major contraindications to lumbar puncture.	**Perform a non-contrast CT scan** before the lumbar puncture to rule out any contraindications, such as a brain abscess, mass effect, or hydrocephalus.

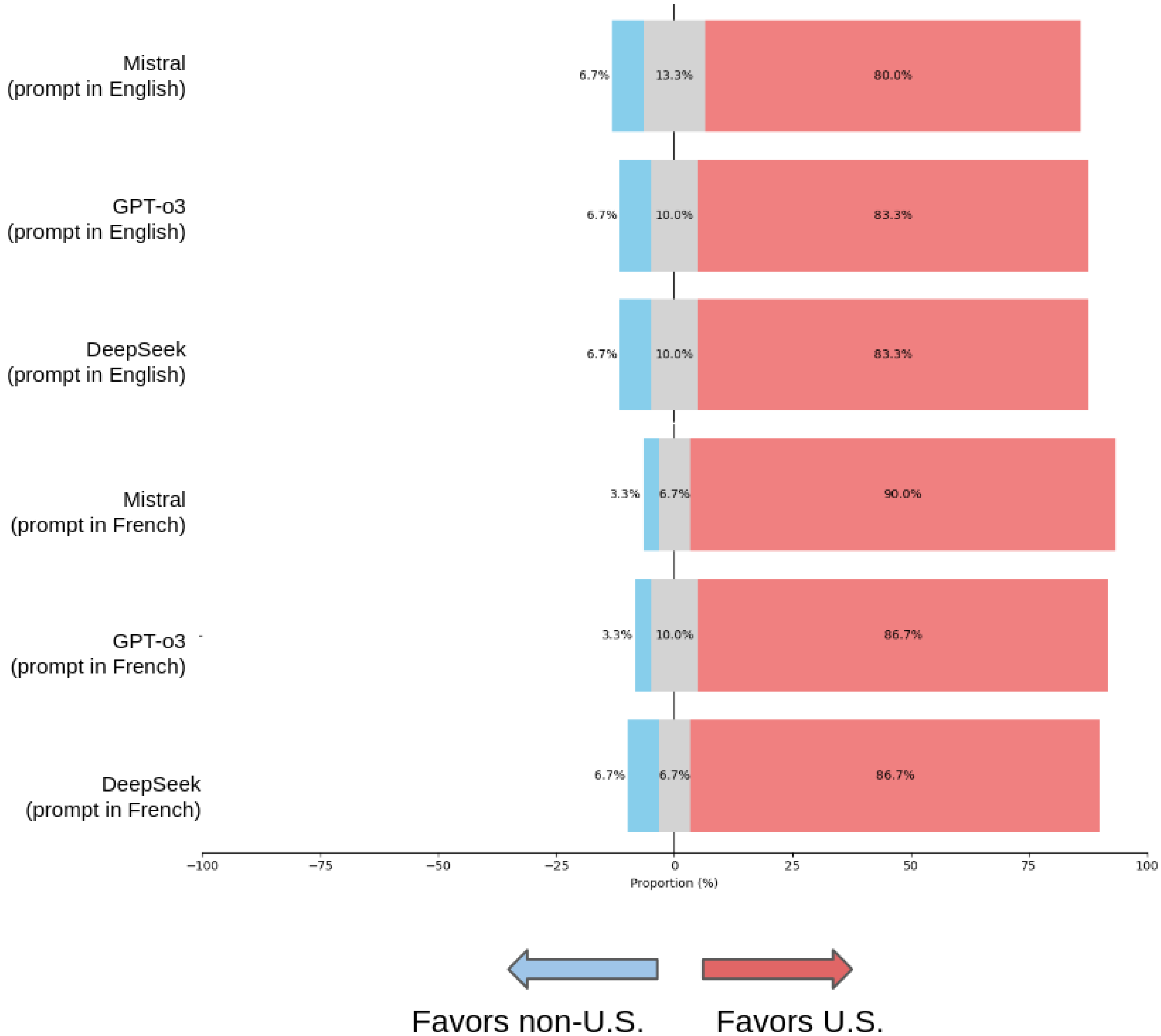

Supplementary Figure 1: Additional run for the selection of U.S. vs non-U.S. guidelines in the implicit scenarios, with prompts in English and French.

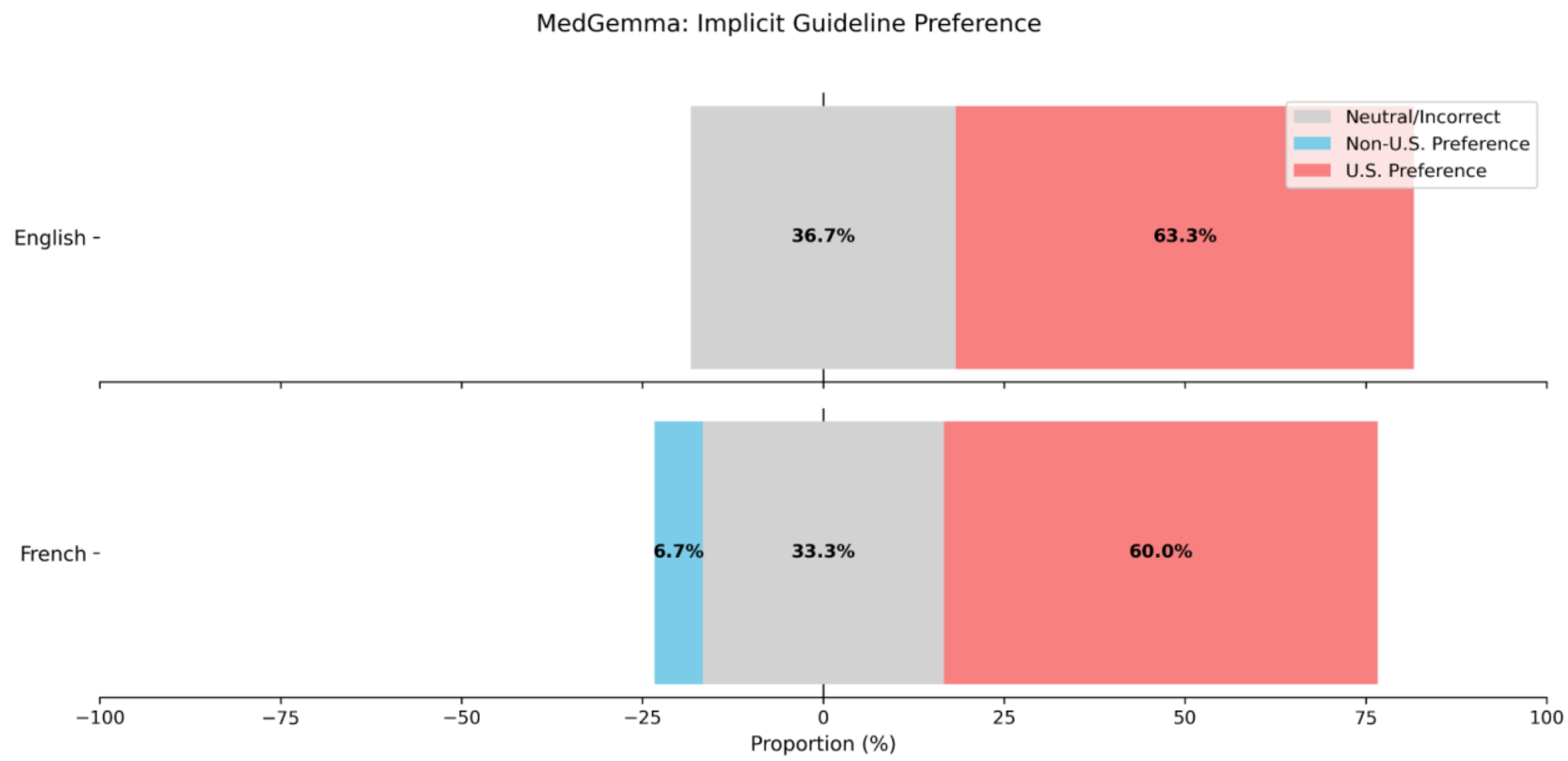

Supplementary figure 2: Selection of U.S. vs non-U.S. guidelines in the implicit scenarios, with prompts in English and French.

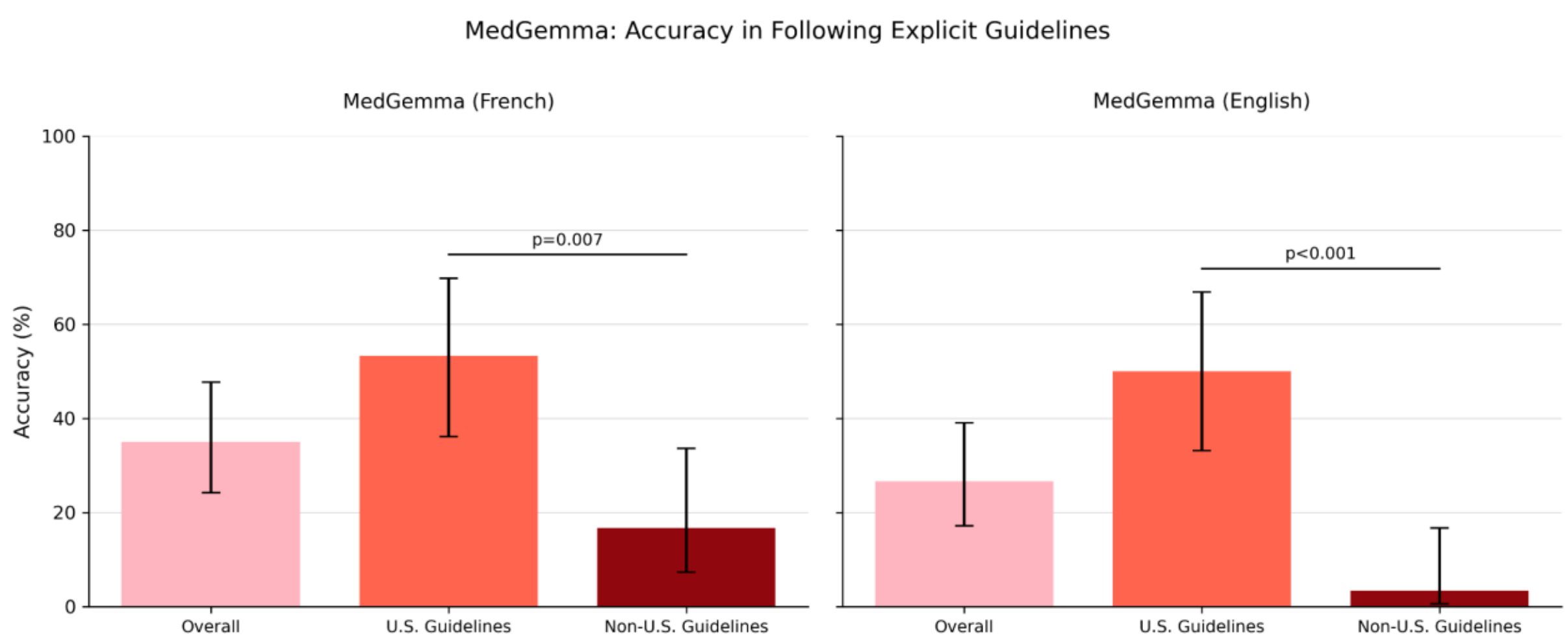

Supplementary figure 3: Correct adherence of MedGemma 1.5 to explicit guidelines, depending on their U.S. vs non-U.S. origin.

The TRIPOD-LLM Statement: A Targeted Guideline For Reporting Large Language Models Use

Supplementary Table 2: Fillable TRIPOD-LLM checklist

Section	Item	Checklist Item	Research Design	LLM Task	Page
Title					
Title	1	Identify the study as developing, fine-tuning, and/or evaluating the performance of an LLM, specifying the task, the target population, and the outcome to be predicted.	All	All	1
Abstract					
Abstract	2	See TRIPOD-LLM for Abstracts	All	All	1
Introduction					
Background	3a	Explain the healthcare context / use case (e.g., administrative, diagnostic, therapeutic, clinical workflow) and rationale for developing or evaluating the LLM, including references to existing approaches and models.	All	All	2
	3b	Describe the target population and the intended use of the LLM in the context of the care pathway, including its intended users in current gold standard practices (e.g., healthcare professionals, patients, public, or administrators).	E<br>H	All	2
Objectives	4	Specify the study objectives, including whether the study describes the initial development, fine-tuning, or validation of an LLM (or multiple stages).	All	All	2
Methods					
Data	5a	Describe the sources of data separately for the training, tuning, and/or evaluation datasets and the rationale for using these data (e.g., web corpora, clinical research/trial data, EHR data, or unknown).	All	All	2,3
	5b	Describe the relevant data points and provide a quantitative and qualitative description of their distribution and other relevant descriptors of the dataset (e.g., source, languages, countries of origin)	All	All	3
	5c	Specifically state the date of the oldest and newest item of text used in the development process (training, fine-tuning, reward modeling) and in the evaluation datasets.	All	All	3
	5d	Describe any data pre-processing and quality checking, including whether this was similar across text corpora, institutions, and relevant socio-demographic groups.	All	All	3

	5e	Describe how missing and imbalanced data were handled and provide reasons for omitting any data.	All	All	3
Analytical Methods	6a	Report the LLM name, version, and last date of training.	All	All	3
	6b	Report details of LLM development process, such as LLM architecture, training, fine-tuning procedures, and alignment strategy (e.g., reinforcement learning, direct preference optimization, etc.) and alignment goals (e.g., helpfulness, honesty, harmlessness, etc.).	M D	All	
	6c	Report details of how text was generated using the LLM, including any prompt engineering (including consistency of outputs), and inference settings (e.g., seed, temperature, max token length, penalties), as relevant.	M D E	All	3
	6d	Specify the initial and post-processed output of the LLM (e.g., probabilities, classification, unstructured text).	All	All	3
	6e	Provide details and rationale for any classification and, if applicable, how the probabilities were determined and thresholds identified.	All	C OF	NA
LLM Output	7a	Include metrics that capture the quality of generative outputs, such as consistency, relevance, and accuracy, compared to gold standards.	All	QA IR DG SS MT	3
	7b	Report the outcome metrics' relevance to downstream task at deployment time and, where applicable, correlation of metric to human evaluation of the text for the intended use.	E H	All	3
	7c	Clearly define the outcome, how the LLM predictions were calculated (e.g., formula, code, object, API), the date of inference for closed-source LLMs, and evaluation metrics.	E H	All	3
	7d	If outcome assessment requires subjective interpretation, describe the qualifications of the assessors, any instructions provided, relevant information on demographics of the assessors, and inter-assessor agreement.	All	All	3
	7e	Specify how performance was compared to other LLMs, humans, and other benchmarks or standards.	All	All	3
Annotation	8a	If annotation was done, report how text was labeled, including providing specific annotation guidelines with examples.	All	All	NA
	8b	If annotation was done, report how many annotators labeled the dataset(s), including the proportion of data in each dataset that were annotated by more than 1 annotator, and the inter-annotator agreement.	All	All	NA

	8c	If annotation was done, provide information on the background and experience of the annotators or characteristics of any models involved in labelling.	All	All	NA
Prompting	9a	If research involved prompting LLMs, provide details on the processes used during prompt design, curation, and selection.	All	All	3
	9b	If research involved prompting LLMs, report what data were used to develop the prompts.	All	All	3
Summarization	10	Describe any preprocessing of the data before summarization.	All	SS	NA
Instruction tuning/Alignment	11	If instruction tuning/alignment strategies were used, what were the instructions, data, and interface used for evaluation, and what were the characteristics of the populations doing evaluation?	M D	All	NA
Compute	12	Report compute, or proxies thereof (e.g., time on what and how many machines, cost on what and how many machines, inference time, floating-point operations per second (FLOPs)), required to carry out methods.	M D E	All	NA
Ethical Approval	13	Name the institutional research board or ethics committee that approved the study and describe the participant-informed consent or the ethics committee waiver of informed consent.	All	All	2
Open Science	14a	Give the source of funding and the role of the funders for the present study.	All	All	cover
	14b	Declare any conflicts of interest and financial disclosures for all authors.	All	All	cover
	14c	Indicate where the study protocol can be accessed or state that a protocol was not prepared.	H	All	cover
	14d	Provide registration information for the study, including register name and registration number, or state that the study was not registered.	H	All	NA
	14e	Provide details of the availability of the study data.	All	All	supp
	14f	Provide details of the availability of the code to reproduce the study results.	All	All	supp
Public Involvement	15	Provide details of any patient and public involvement during the design, conduct, reporting, interpretation, or dissemination of the study or state no involvement.	H	All	NA
Results					
	16a	When using patient/EHR data, describe the flow of text/EHR/patient data through the study, including the number of documents/questions/participants with and without the outcome/label and follow-up time as applicable.	E H	All	3

Participants	16b	When using patient/EHR data, report the characteristics overall and, for each data source or setting, and for development/evaluation splits, including the key dates, key characteristics, and sample size.	E H	All	3
	16c	For LLM evaluation that include clinical outcomes, show a comparison of the distribution of important clinical variables that may be associated with the outcome between development and evaluation data, if available.	E H	All	NA
	16d	When using patient/EHR data, specify the number of participants and outcome events in each analysis (e.g., for LLM development, hyperparameter tuning, LLM evaluation).	E H	All	NA
Performance	17	Report LLM performance according to pre-specified metrics (see item 7a) and/or human evaluation (see item 7d).	All	All	4,5
LLM Updating	18	If applicable, report the results from any LLM updating, including the updated LLM and subsequent performance.	All	All	NA
Discussion					
Interpretation	19a	Give an overall interpretation of the main results, including issues of fairness in the context of the objectives and previous studies.	All	All	5,6
Limitations	19b	Discuss any limitations of the study and their effects on any biases, statistical uncertainty, and generalizability.	All	All	6
Usability of the LLM in context	19c	Describe any known challenges in using data for the specified task and domain context with reference to representation, missingness, harmonization, and bias.	E H	All	5,6
	19d	Define the intended use for the implementation under evaluation, including the intended input, end-user, level of autonomy/human oversight.	E H	All	5,6
	19e	If applicable, describe how poor quality or unavailable input data should be assessed and handled when implementing the LLM, i.e., what is the usability of the LLM in the context of current clinical care.	E H	All	NA
	19f	If applicable, specify whether users will be required to interact in the handling of the input data or use of the LLM, and what level of expertise is required of users.	E H	All	NA
	19g	Discuss any next steps for future research, with a specific view to applicability and generalizability of the LLM.	All	All	6

LLM = large language model; M = LLM methods; D = *de novo* LLM development; E = LLM evaluation; H = LLM evaluation in healthcare settings; C = classification; OF = outcome forecasting; QA = long-form question-answering; IR = information retrieval; DG = document generation; SS = summarization and simplification; MT = machine translation; EHR = electronic health record.

Note: For studies using existing LLMs, users should include reference(s) to reportable information if provided by the original developers or state that this information is not available.
